# Effect of X-ray radiation on the pharmacokinetics of apatinib *in vivo* in rats

**DOI:** 10.3389/fphar.2022.943812

**Published:** 2022-09-12

**Authors:** Shi-Qi Dong, Fan Yang, Dong-Xu Zhang, Ling-Mei Wang, Jian-Feng Liu, Ai-Jie Zhang, Hui-Rong Fan

**Affiliations:** Key Laboratory of Radiopharmacokinetics for Innovative Drugs, Chinese Academy of Medical Sciences and Institute of Radiation Medicine, Chinese Academy of Medical Sciences and Peking Union Medical College, Tianjin, China

**Keywords:** apatinib, pharmacokinetics, X-ray radiation, concurrent chemoradiation therapy, "RT-PK” phenomenon

## Abstract

**Purpose:** The “radiotherapy-pharmacokinetic” (“RT-PK”) phenomenon refers to the fact that radiation can significantly alter the pharmacokinetic behavior of a drug. At present, it is not clear whether there is an “RT-PK” phenomenon that can affect apatinib during concurrent chemoradiotherapy. In this study, we used a rat irradiation model to study the effects of X-ray radiation on absorption, tissue distribution, and excretion of apatinib.

**Method:** Healthy Sprague-Dawley (SD) rats were randomly divided into control and radiation groups. The radiation group was given an appropriate dose of abdominal X-ray radiation, while the control group was not given irradiation. After 24 h of recovery, both groups were given apatinib solution 45 mg/kg by gavage. A quantitative LC-MS/MS method was developed to determine the concentration of apatinib in the rats, so as to compare the differences between the control and radiation groups and thus investigate the modulating effect of radiation on the pharmacokinetics of apatinib in rats.

**Results:** After abdominal X-ray irradiation, the area under the curve (AUC_0-t_) of apatinib in rat plasma decreased by 33.8% and 76.3% at 0.5 and 2 Gy, respectively. Clearance (CL) and volume of distribution (Vd) increased and were positively correlated with radiation dose. X-ray radiation significantly reduced the concentration of apatinib in the liver and small intestine, and there was no tissue accumulation. In excretion studies, we found that X-ray radiation reduced the cumulative excretion of apatinib in feces and urine by 11.24% and 86.17%, respectively.

**Conclusion:** Abdominal X-ray radiation decreased plasma exposure, tissue distribution, and excretion of apatinib in rats, suggesting that the RT-PK phenomenon affects apatinib. We speculate that this RT-PK phenomenon is closely related to changes in metabolic enzymes *in vivo*. In clinical practice, when apatinib is combined with radiotherapy, attention should be paid to adjusting the dose of apatinib and optimizing the treatment plan to alleviate the adverse effects of this RT-PK phenomenon.

## Introduction

Cancer is still a major disease threatening human health, and combined radiotherapy and chemotherapy is recommended as an important treatment option for patients with advanced cancer (Desantis et al., 2014). The conventional application of concurrent chemoradiotherapy causes radiation damage, which is gradually becoming a more prominent and concerning healthcare issue. Radiotherapy changes the physiological state of the body and can even cause pathological changes, which affect the absorption, distribution, metabolism, and excretion of chemotherapeutic drugs in the body. This results in a change in the pharmacokinetic profile of drugs known as the “radiotherapy-pharmacokinetic” (“RT-PK”) phenomenon (Chen et al., 2017; Ishiyama et al., 2019; Hsieh et al., 2020; Liu et al., 2020). Studies have also shown that many drugs can be affected by the RT-PK phenomenon and that radiotherapy significantly changes the pharmacokinetics of many chemotherapeutic drugs, such as 5-fluorouracil, cisplatin, sorafenib, and irinotecan. This not only reduces the efficacy of chemotherapeutic drugs, but even causes more extensive toxic and side effects (Hsieh et al., 2010; Hsieh et al., 2011; Hsieh et al., 2013; Tsai et al., 2015; Qiao et al., 2019; Hu et al., 2020). For example, the area under the curve (AUC) area was reduced by 16% and 29% for 5-fluorouracil and cisplatin, respectively, under 2 Gy head and neck irradiation conditions. Interestingly, different local irradiation modalities at the same irradiation dose may bring about different degrees of effects on drug PK (Hsieh et al., 2013). Another study showed that 2 Gy irradiation of the abdomen reduced AUC of 5-fluorouracil by 31.7% and reduced MRT, Vd, and incremental total plasma clearance as well (Hsieh et al., 2010). RT-PK is becoming a routine phenomenon in clinical radiotherapy and chemotherapy and requires more attention by clinicians and researchers for clinical rational drug use and precise treatment.

Through expert consensus, apatinib, a small molecule tyrosine kinase inhibitor, has become a third-line treatment for advanced gastric cancer (Li et al., 2016). Clinical data show that apatinib is rapidly absorbed after a single dose, with a mean plasma concentration peak time of 1.7–2.3 h and a mean elimination half-life of 7.9–9.4 h; multiple doses over eight consecutive days can achieve a steady state with a half-life of 18.6 h and no significant accumulation. Apatinib has many elimination pathways in the body. Within 96 h after oral administration, the excretion rate of apatinib in feces and urine can reach 69.8% and 7.02%. It is metabolized by phase I and phase II metabolizing enzymes in the human body, thereby transforming into various metabolites, and its biotransformation routes mainly include E- and Z-cyclopentyl-3-hydroxylation, N-dealkylation, pyridyl-25-N-oxidation, 16-hydroxylation, dioxygenation, and O-glucuronidation after 3-hydroxylation (Ding et al., 2013).

In recent years, apatinib combined with radiotherapy has shown good survival benefits in the treatment of non-small cell lung cancer, breast cancer, liver cancer, and cervical cancer (Zhang et al., 2018; Liu et al., 2021; Ma et al., 2021). At present, there have been as many as 25 clinical studies of apatinib combined with radiotherapy, and that number seems to be increasing (http://clinicaltrials.gov/). The combination of apatinib-radiation therapy has been shown to improve patient survival, significantly prolong progression-free survival, and provide good antitumor activity (Hu et al., 2020; Qiu et al., 2022). However, Guo’s study found that, compared with apatinib treatment or radiotherapy treatment alone, patients treated with a combination of apatinib combined with radiotherapy had more extensive side effects, such as proteinuria, hand-foot syndrome, mucositis, etc (Guo et al., 2020). The same results were obtained in the latest study by Hu et al. (2020). How the above-mentioned clinical toxic effects and side effects are related to the RT-PK phenomenon is still unclear, but given that this phenomenon occurs during combined radiotherapy with apatinib therapy, the extent of PK alteration deserves further study.

In this study, X-ray radiation was used as a radiation model in rats and a UPLC-MS/MS method was established to determine the content of apatinib in biological samples of rats in order to systematically explore the effects of X-ray radiation on the absorption, excretion, and tissue distribution of apatinib in rats. The ultimate goal of this study was to provide an experimental and theoretical basis for optimizing treatment plans involving apatinib combined with radiotherapy, adjusting clinical dosing, and carrying out precise treatment.

## Materials and methods

### Reagents and chemicals

Apatinib (purity >98.0%) was purchased from Nanjing Chemlin chemical industry co., LTD. (Nanjing, China). Verapamil (purity >98.0%) was obtained from Raw material medicine reagent co., LTD. (Nanjing, China), which was used as internal standard (IS). The acetonitrile and methanol were HPLC grade and purchased from Fisher Scientific (United States). Ultra-pure water was acquired from Watsons (Hongkong, China). Formic acid (FA) was purchased from DaMao chemical reagent factory (Tianjin, China). Ammonium acetate was obtained from Tianjin Guangfu Technology Development Co., LTD. (Tianjin, China). Other reagents and chemicals were analytical grade. Drug-free heparinized rat plasma was freshly collected from Sprague-Dawley (SD) rats in our laboratory and stored at −20°C before use.

### Instrumentation and conditions

Analysis was performed on an UPLC (Waters, United States) tandem mass spectrometry (Applied Biosystems QTRAP 4500, United States) system. The UPLC system was coupled to mass spectrometer through an electro-spray ionization (ESI) source. The chromatographic separation of apatinib was performed on a Shim-pack Arata C18 column (3.0 × 100 mm, 2.2 μm) at a flow rate of 0.3 ml/min for 5 min. The column temperature was maintained at 40°C during the whole separation process. The mobile phase consisted of acetonitrile (mobile phase A) and 0.1% FA in water containing 1 mmol ammonium acetate (mobile phase B). The initial proportion of gradient was 80% mobile phase B and then held for 0.5 min. The compounds were separated using a linear gradient changing from 80% B to 57% B within 2.7 min. Mobile phase B was then decreasing to 2% over 0.1 min and held for 1 min to clean the column. The gradient ended at the initial conditions for 0.6 min to equilibrate the column. The whole gradient time was 5 min, and the injection volume was set at 1 μl. Quantification was acquired by multiple reaction monitoring (MRM) mode of m/z 398.3→212.2 for apatinib and m/z 455.2→165.2 for IS. The main mass spectrometry parameters were as follows: Curtain Gas 30 psi, Ion Source Gas1 60 psi, Ion Source Gas2 60 psi, Ion Spray Voltage 5000 V, Spray Temperature 550°C. The collision energy for apatinib and IS was 40 and 35 eV, respectively.

The X-ray radiation of rats was delivered by a RS-2000 Pro 225 Biological Irradiator (Rad Source, United States). The experiment animals were exposed to a design dose of abdominal irradiation at a dose rate of 1.090 Gy/min.

### Preparation of calibration curve and quality control samples

Apatinib and verapamil were prepared as a 1 mg/ml stock solution with methanol and the stock solution of apatinib was diluted with methanol to 0.1, 0.2, 1, 2, 4, 10 and 20 μg/ml working solutions and QC was diluted in a similar manner to 0.2, 2 and 16 μg/ml. The stock solution of verapamil was diluted with methanol to a working solution of 200 ng/ml. Both stock and working solutions were stored at 4°C and equilibrated to room temperature before use. 10 μl of apatinib working solution was added to 190 μl blank rat biological samples to make final concentrations of 5, 10, 50, 100, 200, 500 and 1,000 ng/ml. The QC samples were prepared in the same way as the calibration curve, and the final concentrations of LQC, MQC and HQC were 10, 100 and 800 ng/ml, respectively. Both calibration curves and quality control samples were prepared daily to avoid potential degradation or adsorption problems.

### Sample preparation

Biological samples (plasma, tissue homogenates, urine, and fecal homogenates) were thawed at room temperature and vortexed for 10 s, followed by protein precipitation. Take 50 μl of biological sample, add 50 μl of verapamil working solution and 150 μl of precipitant (methanol: acetonitrile, 3:7), vortex for 2 min, centrifuge at 12000 rpm for 10 min at 4°C, and take 100 μl of supernatant to put in the injection vial, 1 μl was quantitatively detected by LC-MS/MS (Dong et al., 2018). The calibration curve and quality control samples were processed as above.

### Method validation

The development of bioanalytical method involved optimizing the procedures and conditions, and the newly developed method should be fully validated to ensure that the method was suitable for application. “M10 Guideline in development on Bioanalytical Method Validation” promulgated by the International Council for Harmonisation of Technical Requirements for Pharmaceuticals for Human Use was the guiding principle for the method validation in this study (https://www.ich.org/page/multidisciplinary-guidelines). The content of method validation mainly included specificity, sensitivity, accuracy, precision, calibration curve, recovery, matrix effects and so on.

To investigate the specificity of the method, the signal-to-noise ratio at the lower limit of quantification (LLOQ) was calculated to assess the sensitivity of the method by analyzing apatinib spiked plasma at LLOQ and the internal standard (six different batches of rat plasma samples). Extraction recoveries of apatinib and IS were evaluated by comparing the peak areas of standards spiked before extraction with those of standards spiked after extraction. The matrix effects of apatinib and IS were assessed by comparing the peak areas of standards with those of standards spiked after extraction. Six replicates of apatinib at four QC levels (5, 10, 100 and 800 ng/ml) were analyzed. Intra-day precision and accuracy were calculated using six replicates (*n* = 6) determinations from six different batches of rat plasma samples during a single analytical run. Inter-day precision and accuracy were calculated using eighteen replicates (*n* = 18) determinations made on three separate days.

### Experimental animals and model preparation

Male SD rats (200 ± 20 g) were purchased from Beijing Weitong Lihua Laboratory Animal Technology Co., Ltd. (Beijing, China) and housed in the SPF animal room of the Institute of Radiation Medicine (CAMS), Chinese Academy of Medical Sciences. All animal experiments followed the regulations of the Animal Ethics Committee of the Institute of Radiation Medicine, Chinese Academy of Medical Sciences. Animals can eat and drink freely, the humidity is 40–60%, the temperature is 22–25°C, 12/12 h alternating light/darkness, and free drinking water but fasting before administration.

Rats were anesthetized with isoflurane and placed on the irradiation table of an RS-2000 Pro 225 Biological Irradiator with an irradiation field setting of 5 cm × 5 cm and dose rates of 1.09 Gy/min, 0.5 Gy (irradiation time, 0.46 min), and 2 Gy (irradiation time, 0.46 min) were delivered. Control group and radiation group animals were transferred and anesthetized simultaneously. Rats were observed for 24 h in the SPF animal room after irradiation, after which absorption, tissue distribution, and urine and fecal excretion studies were performed.

### Apatinib solution preparation

Apatinib Active Pharmaceutical Ingredient was dissolved in glucose injection saline to give the oral administration solution a dose of 45 mg/kg.

### Effect of radiation on plasma exposure of apatinib

Nine SD rats were randomly divided into three groups: 0 Gy, 0.5 and 2 Gy. Rats in each group were given 45 mg/kg of apatinib solution by gavage after 24 h of recovery from different doses of X-ray irradiation. Blood samples were collected predose and at 0.083, 0.25, 0.5, 1, 2, 4, 6, 8 and 12 h postdose in heparinized centrifuge tubes, then centrifuged at 12000 rpm for 10 min at 4°C, and all upper plasma were collected and frozen at −80°C for measurement to compare the differences in exposure levels of apatinib in plasma under different radiation doses.

### Effect of radiation on tissue distribution of apatinib

Twenty-four SD rats were randomly divided into three groups according to time points: 0.25, 1, and 4 h. Each group was further divided into a control group and a radiation group, and the rats in the radiation group were given 2 Gy of single abdominal X-ray irradiation, and after 24 h of recovery, each group was given 45 mg/kg of apatinib by gavage. After the rats were executed at the set time point, the liver and small intestine were taken immediately, washed with 0.9% saline and blotted with filter paper. About 200 mg of tissues were weighed into a centrifuge tube, 4 times the volume of homogenizing solvent (50% methanol water) was added, and the tissues were ground and homogenized in a homogenizer, and all the tissues were collected and stored at −80°C, and to investigate the effect of X-ray radiation on the tissue distribution of apatinib.

### Effect of radiation on urine and fecal excretion of apatinib

Six SD rats were randomly divided into control and radiated groups. The rats in the radiated group were given a single dose of 2 Gy abdominal X-ray radiation, and after 24 h of recovery, apatinib was administered by gavage at a dose of 45 mg/kg in both the radiated and control groups. urine samples were collected before and 4, 12, 24, 48, and 72 h after administration (kept frozen throughout the collection period) and the volumes were recorded. Stool samples before (0 h) and 4, 8, 12, 24, 48 and 72 h after administration were collected and weighed, and ground into a homogenate by adding 4 times the volume of 50% methanolic water.

## Data analysis

Apatinib was quantitatively analyzed using Analyst 1.6.3 software. A test of normality, *t*-test, and One-way ANOVA were performed using SPSS 17.0 with post-hoc multiple comparisons and the LSD method, and the difference was considered to be statistically significant when *p* < 0.05. The results are presented as means ± standard deviations. GraphPad Prism 8.0.1 software was used for graphing.

Pharmacokinetic parameters were calculated using WinNonlin 8.1 software and a non-compartmental method, with three significant digits retained. The area under the concentration-time curve (AUC) was calculated by use of the log-linear trapezoidal rule. Clearance was calculated by dividing the dose by the AUC0-t. The rate constant K is derived from the end of the blood concentration-time curve to obtain the half-life. The Volume of distribution was determined by dividing the Clearance by the rate constant K.

## Result

### Method validation

**FIGURE 1:**
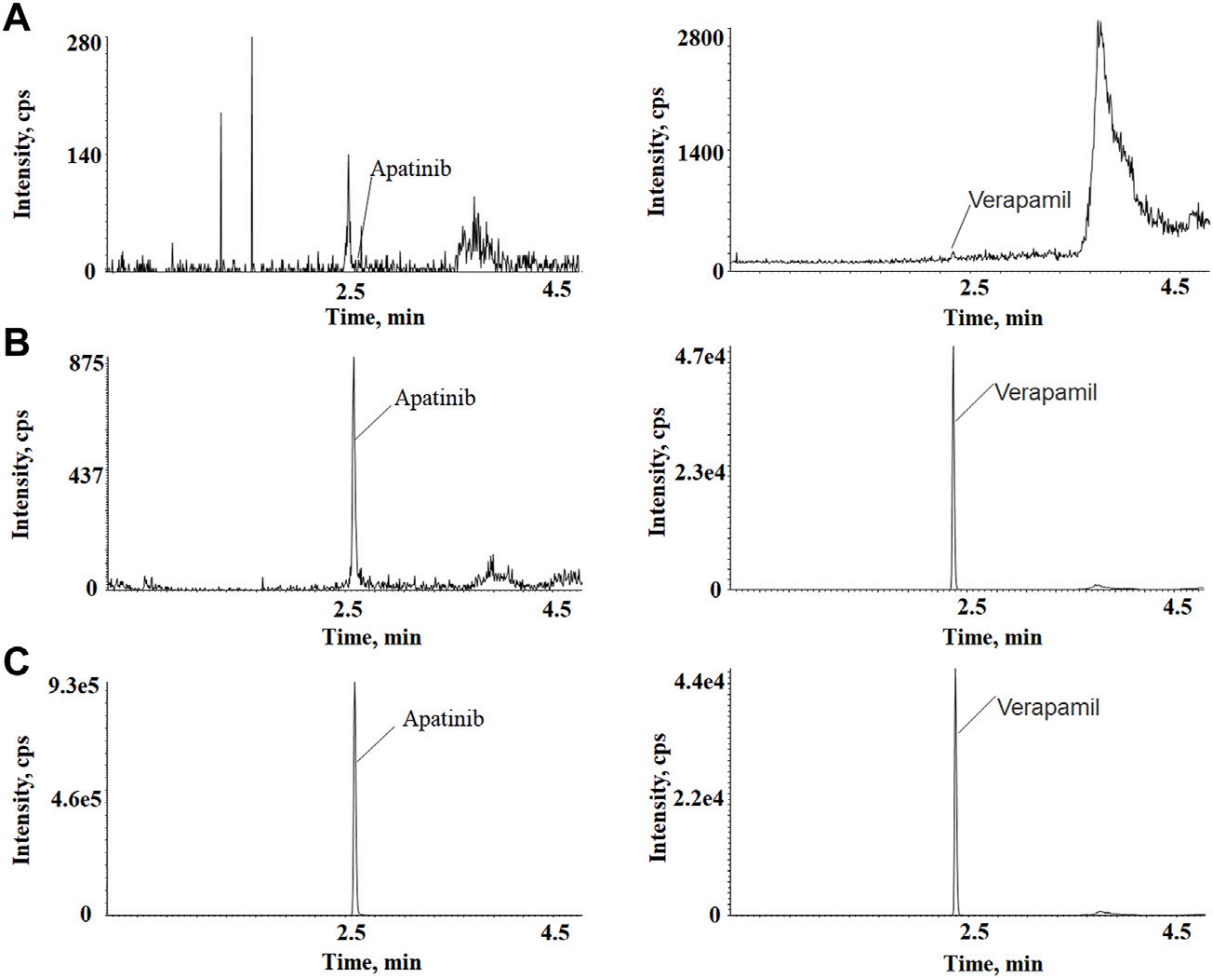
**(A)** Blank rat plasma sample. **(B)** Blank plasma sample spiked with 5 ng/ml of apatinib and 200 ng/ml of IS. **(C)** A plasma sample collected at 3 h after intragastrical administration of.

The representative chromatograms of apatinib and verapamil used to evaluate sensitivity and specificity are shown in Figure 1, including a blank plasma sample, an LLOQ sample, and an unknown plasma sample obtained 3 h after intragastric administration. There were no interfering peaks of analytes at the retention time in the blank sample. The response intensity of the LLOQ sample (5 ng/ml) met the acceptance criteria. The calibration curve for apatinib was solidly linear in the concentration range of 5–1,000 ng/ml, with a calibration curve of y = 0.00348x + 0.00265, and *R* ^2^ > 0.99. In this study, the precision and accuracy of the four concentrations were investigated separately, as shown in Table 1. The intra-day and inter-day precision of apatinib ranged from 2.32 to 6.49% and 3.11–8.26%, respectively. The intra-day and inter-day accuracies of apatinib were −15.3–4.67% and −9.02–3.64%, respectively. The ion enhancement or suppression of analytes in biological samples is expressed as a matrix effect, which should be close to 100% by optimizing the experimental conditions to avoid endogenous interference to the assay results. The matrix effects at concentrations of LQC and HQC were (109 ± 13)% and (102 ± 2.4)% for apatinib and (106 ± 2.6)% for IS, indicating that the matrix had no effect on the results of the assay. The recovery was evaluated at the same concentration as the matrix and showed (96.1 ± 3.0)% and (102 ± 3.2)%, respectively. The recovery of verapamil was (108 ± 3.4)%. Stability studies showed that apatinib was stable under a variety of storage conditions (Lou et al., 2016).

**TABLE 1:** Intra- and inter-day accuracy and precision of the method for the determination of apatinib in rat plasma (Mean, *n* = 6).

Spiked concentration (ng/ml)	Accuracy (RE %)				Precision (CV %)			
	Intra-day			Inter-day	Intra-day			Inter-day
	Day 1	Day 2	Day 3		Day 1	Day 2	Day 3	
5	−15.3	−10.0	−1.33	−8.89	3.86	5.07	6.49	8.26
10	−5.00	−4.67	4.67	−1.67	4.37	5.38	4.82	6.55
100	2.43	4.23	4.25	3.64	2.38	2.59	4.22	3.11
800	−11.5	−8.58	−6.96	−9.02	2.32	5.37	2.85	4.13

The intra-day and inter-day precision of apatinib were in the range of 2.32–6.49% and 3.11–8.26%, respectively. The intra-day and inter-day accuracy of apatinib were in the range of -15.3–4.67% and -9.02–3.64%, respectively.

### Effect of radiation on plasma exposure of apatinib

**FIGURE 2:**
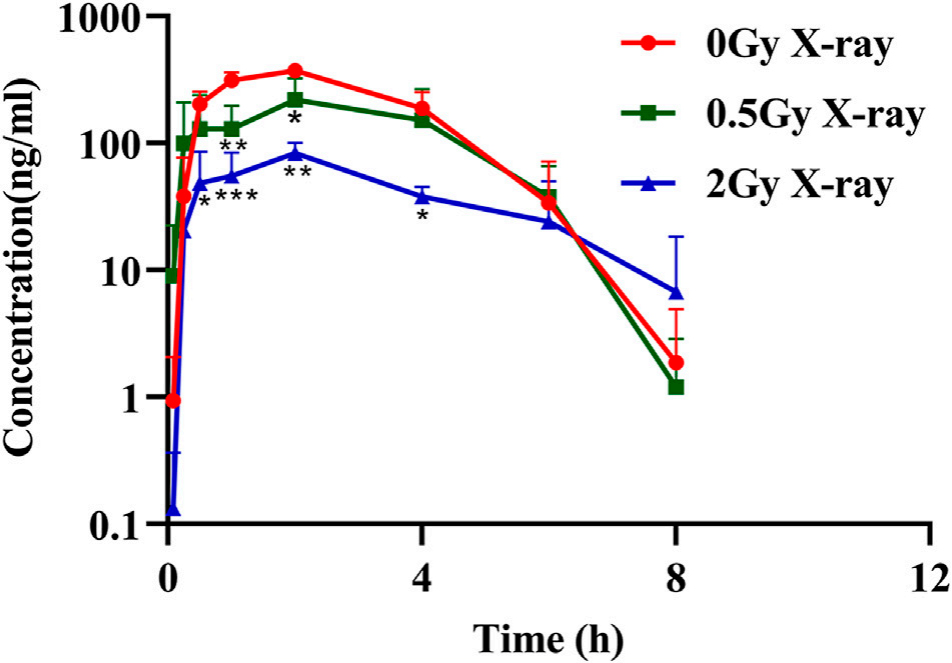
The mean plasma concentration–time profile of apatinib in rat plasma presence or absence abdominal X-ray irradiation after intragastrical administration of 45 mg/kg (Mean ± SD, *n* = 3). * significantly different from the absence X-ray irradiation group at *p* < 0.05, ** significantly different from the absence X-ray irradiation group at *p* < 0.01, *** significantly different from the absence X-ray irradiation group at *p* < 0.001.

The effects of different doses of X-ray radiation on the plasma AUC_0-t_ of rats are shown in Figure 2 and the results of pharmacokinetic parameters are shown in Table 2. Compared with the control group, the AUC_0-t_ of apatinib in rat plasma was reduced after abdominal X-ray irradiation by 33.8% and 76.3% at 0.5 and 2 Gy, respectively. However, there was no statistically significant difference at 0.5 Gy (*p* = 0.065), while there was a significant difference at 2 Gy (*p* < 0.01). The trend of the peak concentration (C_max_) was basically consistent with the AUC_0-t_. In contrast, X-ray radiation increased the clearance (CL) of apatinib by 1.75-fold (0.5 Gy) and 3.65-fold (2 Gy). Compared with the control group, the apparent volume of distribution increased by 2.46-fold (0.5 Gy) and 13.12-fold (2 Gy), respectively, while the mean residence time (MRT) of the two groups was not significantly different. In conclusion, the results of these tests showed that radiation decreased plasma exposure to apatinib, with the trend being clearer at higher radiation doses. This study preliminarily demonstrates that the RT-PK phenomenon can affect apatinib.

**TABLE 2:** Pharmacokinetic parameters of apatinib (45 mg/kg, intragastrical administration) in rat plasma after abdominal X-ray irradiation (Mean ± SD, *n* = 3).

Parameter		0 Gy	0.5 Gy	2 Gy
T_1/2_	h	0.548 ± 0.162	0.776 ± 0.450	2.21 ± 2.18
C_max_	μg/mL	0.374 ± 0.0307	0.243 ± 0.101^*^	0.0833 ± 0.0171^**^
T_max_	h	1.67 ± 0.577	1.50 ± 0.866	2.00 ± 0.00
AUC_0-t_	μg·h/mL	1.32 ± 0.154	0.874 ± 0.391	0.313 ± 0.0129^**^
AUC_0-∞_	μg·h/mL	1.33 ± 0.154	0.878 ± 0.388	0.369 ± 0.0755^**^
Vd	L/kg	0.0267 ± 0.00584	0.0657 ± 0.0376	0.350 ± 0.286
CL	L/h/kg	0.0343 ± 0.00391	0.0600 ± 0.0306	0.125 ± 0.0245^**^
MRT	h	2.47 ± 0.372	2.71 ± 0.325	2.90 ± 1.09

T_1/2_, Elimination half-life; C_max_, The peak plasma concentration; T_max_, Time to reach C_max_; AUC_0-t_, Area under the concentration-time curve from 0 to 12 h; AUC_0-∞_, Area under the curve from 0 to infinity; Vd, Volume of distribution; CL, clearance; MRT, Mean residence time. * significantly different from the absence X-ray irradiation group at *p* < 0.05, ** significantly different from the absence X-ray irradiation group.

### Effect of radiation on tissue distribution of apatinib

**FIGURE 3:**
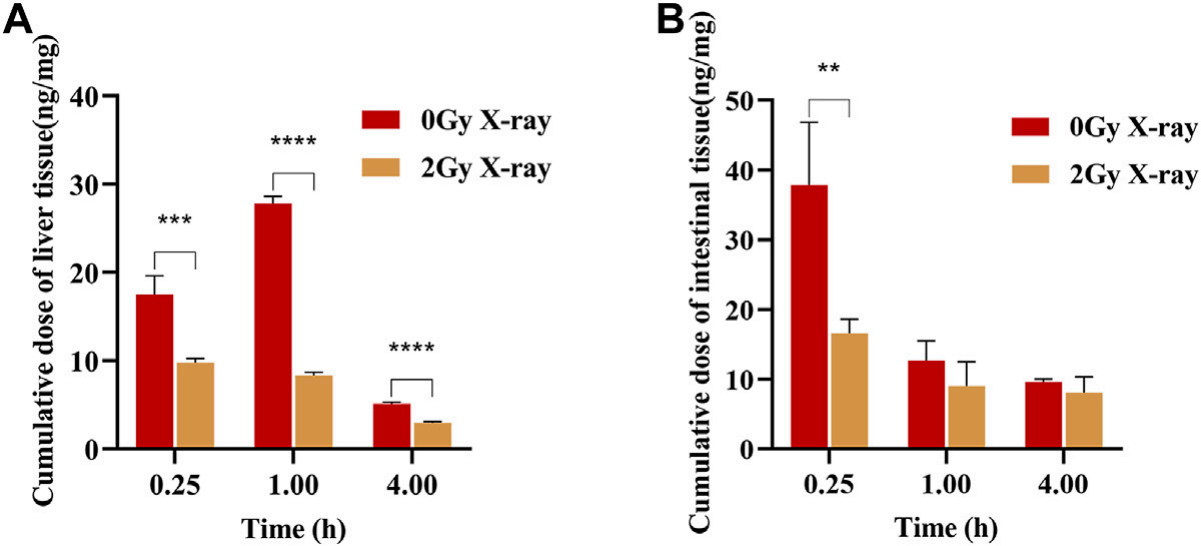
Apatinib accumulation in liver **(A)** and small intestine **(B)** at 0.25, 1, 4 h after intragastric administration presence or absence abdominal X-ray irradiation 45 mg/kg (Mean ± SD, *n* = 4). The *t*-test was used for data analysis between the two groups. ** significantly different from the absence X-ray irradiation group at *p* < 0.01, *** significantly different from the absence X-ray irradiation group at *p* < 0.001, **** significantly different from the absence X-ray irradiation group at *p* < 0.0001.

The main distribution organs of apatinib *in vivo* are the liver and the small intestine, so this study focused on the effect of abdominal X-ray radiation on the distribution of apatinib in rat livers and small intestines (Feng et al., 2018). The distribution of apatinib in livers and small intestines after intragastric administration of apatinib under different radiation conditions is shown in Figure 3. The results showed that X-ray radiation could significantly reduce the concentration of apatinib in the liver compared with the control group, and that the difference was statistically significant (*p* < 0.001). In the small intestine, X-ray radiation also caused a decrease in apatinib drug concentration. While the difference between 1 h (*p* = 0.157) and 4 h (*p* = 0.246) groups was not statistically significant, there was a significant difference in the 0.25 h group (*p* < 0.01).

### Effect of radiation on urine and fecal excretion of apatinib

**FIGURE 4:**
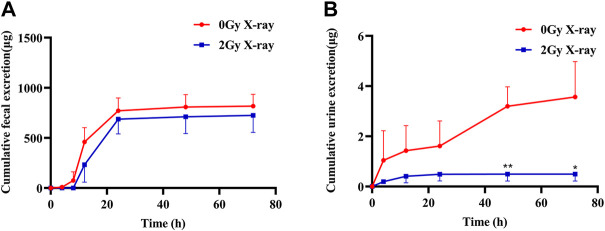
The mean cumulative fecal **(A)** and urine **(B)** excretion of apatinib in rat presence or absence abdominal X-ray irradiation after intragastrical administration of 45 mg/kg (Mean ± SD, *n* = 3). The *t*-test was used for data analysis between the two groups. * significantly different from the absence X-ray irradiation group at *p* < 0.05, ** significantly different from the absence X-ray irradiation group at *p* < 0.01.

As shown in Figure 4, the cumulative excretion of apatinib in feces and urine was 817.2 and 3.6 μg, respectively, after the rats in the control group were given apatinib by gavage, indicating that the main excretion route of apatinib was fecal excretion, with only a very small amount of the drug excreted in the urine. Compared with the control group, abdominal X-ray radiation reduced the cumulative excretion of apatinib in feces and urine by 11.24% and 86.17%, respectively, suggesting that radiation may promote biotransformation of apatinib in the liver, with reduced excretion of the prototype drug and its metabolites.

## Discussion

Concurrent chemoradiotherapy (CCRT) is becoming a standard treatment strategy for various malignancies, particularly locally advanced cancers. It is estimated that more than half of cancer patients receive CCRT during treatment the course of treatment (Bray et al., 2018). CCRT can bring added survival benefits to cancer patients, such as prolonging progression-free survival and increased overall survival (Schoell et al., 1999; Pan et al., 2018; Yang et al., 2019; Hu et al., 2020). However, CCRT is a double-edged sword. A large amount of clinical data show that patients with CCRT have a wider range of toxic and side effects, which should not be ignored (https://pubmed.ncbi.nlm.nih.gov/). The Hsieh study found that radiation significantly reduced the exposure of 5-fluorouracil and its metabolites *in vivo* and altered their pharmacokinetic profiles. Compared to radiotherapy or chemotherapy alone, 5-fluorouracil combined with radiotherapy leads to serious hematological and gastrointestinal reactions (Chen et al., 2017; Liu et al., 2020). The incidence of adverse reactions of sorafenib in combination with radiotherapy exceeds 30%, and there is evidence that the occurrence of toxic and side effects is closely related to the changes in the pharmacokinetics of sorafenib (Hsieh et al., 2020; Tsai et al., 2021). The phenomenon of “RT-PK” is becoming common and is an important limiting factor in CCRT. Therefore, it is very necessary and imperative to conduct extensive RT-PK research in the course of clinical CCRT.

Apatinib is a small molecular tyrosine kinase inhibitor, which accomplishes an anti-tumor effect by inhibiting tumor angiogenesis (Tian et al., 2011; Geng and Li, 2015). In recent years, a large number of studies have found that apatinib combined with radiotherapy has shown good efficacy in the treatment of many cancers. However, there is no relevant report on whether apatinib results in the RT-PK phenomenon in CCRT. In order to solve this problem, we studied the effect of X-ray radiation on apatinib systematically. We have established a fast, sensitive, and reliable LC-MS/MS method for the quantification of apatinib in biological samples, with complete methodological validation. By optimizing the chromatographic and mass spectrometric conditions, we found that the accuracy of our methods was in the range of 5–1,000 ng/ml (*R* ^2^ > 0.99) without matrix interference and the extraction recovery met the experimental requirements. The method validation results showed that the analytical method was reproducible and reliable, which will provide methodological support for further in-depth study of apatinib.

In the pharmacokinetic study of apatinib, we used radiation doses of 0.5 and 2 Gy to simulate the daily treatment dose of tumor patients, which represent the non-target and target doses, respectively (Hsieh et al., 2010; Hsieh et al., 2011). Results showed that X-ray radiation significantly decreased the exposure of apatinib in rats plasma, suggesting that there may be an RT-PK phenomenon affecting apatinib in CCRT. The degree of alteration of apatinib pharmacokinetics was significantly higher at a radiation dose of 2 Gy than that at 0.5 Gy, suggesting that the RT-PK phenomenon of apatinib has a certain dose-dependency. Figure 2 showed that there was a difference in the elimination of oral apatinib, with a significantly faster elimination rate (ke) in the 2 Gy radiation group, suggesting that a flip-flop phenomenon may occur at 2 Gy (Yáñez et al., 2011). In fact, under the conditions of abdominal irradiation, it is inevitable that the intestine in the radiation field will be damaged, and this damage may lead to adverse consequences (Kumagai et al., 2018). Therefore, we speculate that this flip-flop phenomenon may be due to the slow absorption caused by X-ray radiation impairing intestinal function, which is subject to further study. In addition, AUC_0-t_ and C_max_ decreased significantly, while CL increased significantly, indicating that radiation can accelerate the elimination of apatinib *in vivo*, a process that may be closely related to the increase of excretion or metabolic transformation. Compared with the control group, the Vd value of the radiation group increased, although there was no significant difference (*p* = 0.784 (0.5 Gy), 0.055 (2 Gy)). This suggests that radiation may lead to the accumulation of drugs in the tissue.

Considering that the main distribution organs of apatinib are the liver and small intestine (Feng et al., 2018), we focused on these organs in this tissue distribution study. The results showed that the concentration of apatinib in the liver and small intestine of the radiation group was significantly lower than that of the control group, suggesting that X-ray radiation could reduce the tissue distribution of apatinib. Although there was a trend of increasing Vd values, the Vd in rats was much smaller than the total body fluid volume (approximately 0.167 L in 0.25 kg rats, i.e., 0.668 L/kg), suggesting that apatinib was mainly distributed between the total body fluid and plasma volume and mostly unbound to tissues (Davies and Morris., 1993; Wei and Zhao., 2008). However, this study only examined the distribution of apatinib in enterohepatic tissues and not in other tissues. Therefore, the trend of increasing Vd does not necessarily indicate an increase in enterohepatic tissues. The reduction in plasma exposure and tissue distribution of apatinib suggested, to some extent, that radiation may accelerate the elimination of apatinib *in vivo*, such as through excretion or biotransformation, and therefore further excretion studies were performed. In the excretion study, we found that X-ray radiation reduced the cumulative excretion of apatinib in feces and urine by 11.24% and 86.17%, respectively. The decrease in urinary excretion may be due to a decrease in exposure of apatinib to the blood, with this decrease in plasma exposure allowing for a decrease in the filtration rate of the kidneys, which then reduces the renal excretion of apatinib. Although radiation has a significant effect on the excretion of apatinib in urine, urine is the secondary excretion route of drugs, and only a small amount of apatinib is excreted through urine. Therefore, the total excretion of apatinib in urine and feces in the radiation group was slightly lower than that in the control group, but there was no significant difference between the two groups (*p* = 0.472). The above speculation and the results of excretion experiments together suggest that radiation may accelerate the biotransformation of apatinib in rats and that the radiation-induced acceleration of metabolism may have affected the clearance of apatinib. The experimental results of absorption, tissue distribution, and excretion showed that X-ray radiation could significantly change the pharmacokinetic characteristics of apatinib in rats, demonstrating an RT-PK phenomenon resulting from concurrent apatinib and radiotherapy.

In the above studies, X-ray radiation decreased the drug concentration of apatinib to varying degrees, indicating that abdominal X-ray radiation may lead to extensive metabolic transformation of apatinib in the body. Qiao et al. found that the exposure of irinotecan in rats was significantly decreased after 5 Gy X-ray radiation, and the clearance rate was increased by 60% compared with the control group. Further studies found that the expression of metabolic enzymes CYP3A4 and CES1, which mediate irinotecan metabolism, were significantly up-regulated, suggesting that X-ray treatment accelerates the transformation of irinotecan by inducing the expression of metabolic enzymes (Qiao et al., 2019). High-dose X-ray treatment could also significantly increase the protein and mRNA expression levels of CYP1A2 and CYP2E1 in rats, as well as the activities of metabolic enzymes (Li et al., 2020). The body changes caused by irradiation are complex and diverse, and metabolic enzymes undoubtedly play an important role in the regulation of pharmacokinetics. We speculate that the RT-PK phenomenon associated with apatinib is closely related to changes of metabolic enzymes in the body. However, the effects of radiation on the metabolic pathway of apatinib and the regulation of metabolic enzymes need to be studied further. Additionally, the molecular mechanism of the interaction between radiation and chemotherapeutic drugs is not clear. It is therefore necessary to integrate multidisciplinary research and technologies to explain the mechanism of the RT-PK phenomenon at the level of gene regulation and cellular signal pathway.

## Conclusion

The LC-MS/MS method established in this study can accurately quantify the concentration of apatinib *in vivo*. Using this method, we evaluated the effects of X-ray radiation on the absorption, tissue distribution, and excretion of apatinib for the first time. Our study found that apatinib clearly showed an RT-PK phenomenon under the conditions of X-ray radiation, and the effect of radiation on the pharmacokinetics of apatinib was dose-dependent (the higher the radiation dose, the more significant the change in pharmacokinetics). X-ray radiation was able to significantly reduce the plasma exposure, tissue distribution, and excretion of apatinib in rats, and the accelerated metabolism of apatinib induced by radiation may even be the direct cause of the RT-PK phenomenon. Further study on the metabolism of apatinib will contribute to a more comprehensive and in-depth understanding of RT-PK phenomenon. This study provides an experimental and theoretical basis for optimizing treatment plans using apatinib combined with radiotherapy, adjusting clinical drug dose, and enacting accurate treatment to effectively alleviate the occurrence of the RT-PK phenomenon.

## Data Availability

The original contributions presented in the study are included in the article/supplementary material, further inquiries can be directed to the corresponding authors.
